# Approaches for the Elimination of Microbial Contaminants from *Lippia multiflora* Mold. Leaves Intended for Tea Bagging and Evaluation of Formulation

**DOI:** 10.1155/2022/7235489

**Published:** 2022-02-27

**Authors:** Doris Kumadoh, Mary-Ann Archer, Michael O. Kyene, Genevieve N. Yeboah, Ofosua Adi-Dako, Christina Osei-Asare, Emmanuel Adase, Susana Oteng Mintah, Hilda Amekyeh, Alfred A. Appiah

**Affiliations:** ^1^Department of Pharmaceutics, Centre for Plant Medicine Research, Mampong-Akuapem, Ghana; ^2^Department of Production, Centre for Plant Medicine Research, Mampong-Akuapem, Ghana; ^3^Department of Pharmaceutics and Microbiology, School of Pharmacy, University of Ghana, Legon, Accra, Ghana; ^4^Department of Pharmaceutics and Microbiology, Central University College, Accra, Ghana; ^5^Department of Microbiology, Centre for Plant Medicine Research, Mampong-Akuapem, Ghana; ^6^Department of Pharmaceutics, School of Pharmacy, University of Health and Allied Sciences, Ho, Ghana; ^7^Department of Phytochemistry, Centre for Plant Medicine Research, Mampong-Akuapem, Ghana

## Abstract

Elimination of microorganisms from herbal products has been a major concern due to its implicated health risk to consumers. Drying of herbal materials has been employed for centuries to reduce the risk of contamination and spoilage. The present study adopted three drying approaches in an attempt to eliminate microorganisms from *Lippia multiflora* tea bag formulation. This study also evaluated the tea bags and optimized the extraction procedure. The *L. multiflora* leaves for tea bagging were air-dried and milled (A), oven-dried and milled (B), and microwaved (the milled air-dried leaves) (C). The moisture contents were determined at 105°C ± 2°C for 2 hours to constant weight. Phytochemical parameters such as phytochemical constituents, total water extractive, and pH were assessed. The microbial safety and quality of the *L. multiflora* tea bags were evaluated using the British Pharmacopoeia 2019 specifications. The uniformity of the mass of the formulated tea bags was also determined. Extraction from the *Lippia* tea bags was optimized. The results showed that using the approaches (A, B, and C) adopted for drying and processing, the moisture contents of the formulated tea bags were in the range of 9.75–10.71% ^w^/_w_. All the formulated tea bags contained reducing sugars, phenolic compounds, polyuronides, flavonoids, anthracenosides, alkaloids, saponins, and phytosterols. The pH range of the formulations was 7.11–7.54, whereas the total water extractive values were in the range of 19.12–20.41% ^w^/_w_. The one-way analysis of variance demonstrated no significant difference in the data obtained from the results from A, B, and C. The formulation from A was found to be unsafe for consumption due to unacceptable microbial contamination limits. Microbial load of the formulations from B and C were within the BP specifications. All the batches of the formulations passed the uniformity of mass test. An optimized extraction procedure was obtained when one tea bag was extracted in 250 mL of hot water within the specified time. *L. multiflora* leaves meant for tea bagging should be oven-dried or microwaved before tea bagging for safe consumption.

## 1. Introduction

West Africa is endowed with many stouts, woody and aromatic perennial shrubs, of which *Lippia multiflora* Moldenke is a typical example. *L. multiflora* belongs to the Verbenaceae family and grows up to a height of 2.8–4 m [1]. *L. multiflora* is normally found in the forest savanna, sub-savanna, and transitional areas of Ghana. The leaves of *L. multiflora* have been formulated into a herbal tea that is used because of its aromatic odor and therapeutic property of stress relief [2, 3]. The leaves of *L. multiflora* are normally used for the management of mild hypertension and as a diuretic [4, 5]. Studies on the anti-inflammatory, antimalarial, antioxidant [6], analgesic, and antipyretic activities of the *L. multiflora* have been conducted justifying its traditional use for the related conditions [4]. The determination of the shelf life of *L. multiflora* leaves (*Lippia* tea) indicated a period of 62.97 months as the shelf life in a study conducted by Kumadoh and colleagues when the product was stored at specified temperatures of 30°C ± 2°C/70% ± 5% RH (relative humidity) [7]. A study on the safety of a powder prepared from the dried leaves of *L. multiflora* has also been reported by Djengue and colleagues [8]. Results obtained from this study showed that the powder displayed microbial safety at the beginning of storage until at least 18 months of storage without risk of contamination for the consumer. However, powders bought from the markets could not be stored for more than 12 months due to pathogenic and flora contaminations [8].

The presence of microbial contaminants in some herbal products may pose a public health risk to consumers [9, 10] with concerns about microbial contamination in herbal products being a major reason why some consumers avoid herbal products in favor of orthodox medicines [11]. When plant material processing strategies such as harvesting, drying, milling, tea bagging, packaging, storage, and distribution are not properly done by following good manufacturing practices, microbial contamination may result [12, 13]. Some of the ways by which microbial contamination of food products can be eliminated include efficient drying, chilling, curing, and freezing [14]. Irradiation, sterilization, and pasteurization could also make microorganisms inactive for growth and multiplication, thereby preventing contamination. In addition, steam sterilization and ozonation have been reported to be useful in controlling microbial growth in herbs [15]. Studies have shown *L. multiflora* microbial contamination could be reduced by steam treatment [16]. Some factors that should be considered when selecting methods for microbial decontamination include safety, quality, efficacy, practicability, and cost. Care must be taken to prevent the deterioration of essential constituents such as antioxidants and other phytochemical compounds in plant materials during the microbial decontamination process [16]. Microorganisms contaminate various parts of herbal plants, including leaves, stems, flowers, seeds, and roots [17, 18]. It is important to eliminate microbial contaminants in the herbal tea of *L. multiflora* in order to ensure its safety and quality. The aim of this study is to develop a simple and practical method for the elimination of microbial contaminants from herbal tea without adversely affecting the phytochemical constituents and quality properties of the tea. An attempt has also been made to do an optimization of the extraction process for the formulated tea bags.

## 2. Materials and Methods

### 2.1. Plant Material Collection

Fresh leaves of *L. multiflora* were harvested from the Centre for Plant Medicine Research (CPMR) farm at Mampong-Akuapem, Ghana (5°55′ 06.6″ N, 0° 07′57′57 W) and authenticated by the Plant Development Department, CPMR. The plant specimen has been placed in its herbarium with the identification code CPMR 5073. The methods that were used for processing the materials before tea bagging have been detailed below.

### 2.2. Plant Material Processing

Three different methods were used for the processing of the plant material. For each method, four different batches of tea bags were formulated for subsequent analyses. The tea bags manufactured were batch coded as LTB1, LTB2, LTB3, and LTB4, respectively.

#### 2.2.1. Methods of Processing: Air-Drying, Oven-Drying, and Microwaving of *L. multiflora* Mold. Leaves

The method of processing the fresh leaves of *L. multiflora* was modified from Mahanom et al [19], Roshanak et al. [20], and Manzano Santana et al. [21]. The fresh leaves of *L. multiflora* were washed in 70% (^v^/_v_) ethanol and subsequently washed with water to remove all residual ethanol, dust, and unwanted materials accumulated on the leaves. The leaves were air-dried for 7 days on wooden-structured shelves with their bottoms covered with a wire mesh in a well-ventilated drying room for the air-drying method. In the oven-drying method, the leaves, after being washed, were dried in an oven (IGNIS, Italy) at a temperature of 70°C for 2 hours. Dried leaves obtained using the two methods were then milled to obtain a coarse powder (ca 2 mm^2^ in size) using a stainless-steel hammer mill (JICA, Japan) machine and sieved using a 2 mm mesh sieve. The third method of processing involved microwaving of a sample of the milled plant material obtained from the air-drying method for 5 minutes. Tea bagging of the material was done using a tea-bagging machine (GATBPM, India). The obtained tea bags were packaged in ziplock bags and placed in a box. The procedure was repeated for four different batches of the product for each processing method.

### 2.3. Determination of Moisture Content of Powder for Tea Bagging

The moisture contents (percentage) of 3 g of milled material obtained from each processing method which were to be used in the preparation of the tea bags were determined using a moisture content analyzer (Kern, MLB 50-3, Germany) at a temperature of 105°C ± 2°C for 2 h to constant weight. The moisture content was deduced as the percentage of the lost weight divided by the initial weight. This was determined in triplicate for each method and the mean percentage moisture content calculated [22].

### 2.4. Phytochemical Analysis

The formulated tea bags were assessed for phytochemical parameters such as phytochemical constituents, total water extractive, and pH. Standard phytochemical screening tests were used to detect secondary metabolites from *L. multiflora*. Mayer's tests for alkaloids, Fehling's test for free reducing sugars, the Liebermann–Burchard test for phytosterol and triterpenes, froth test for saponins, Shinoda's test for flavonoids, Borntrager's test for free anthracenosides, and ferric chloride solution test for phenolics were used [23].

### 2.5. Determination of pH

A pH meter was calibrated with standard pH buffer solutions beginning with buffers of pH 7 and 10 (Riedel-de Haen, Germany) followed by that with pH 4 (Reagacon, Ireland) to determine the linearity of response of the electrode. To determine the pH, a cup was filled with a portion of a 1%w/v infusion prepared from the tea bags to obtain a preliminary value. Subsequent readings were taken on additional portions of the same sample to yield more constant pH values that were reproducible to ±0.04 units and that showed drifts of less than ±0.04 unit in 2 min, and the maximum pH value obtained recorded. The procedure was done in triplicate for all four batches of products from the three processing methods [24].

### 2.6. Determination of Total Water Extractive

One gram of the material in a *Lippia tea* bag was weighed into a 250 mL flask with a stopper. Next, 100 mL of distilled water was added and the mixture was placed on a water bath for 1 hour. The extract was filtered using Johnson test paper (Qualitative filter paper, grade 304; 125 mm) and 10 mL of the filtrate was pipetted for drying in an evaporating dish at 105°C for 1 h to a constant weight. The evaporating dish containing the residue was transferred into a desiccator to cool and later weighed [25].

The weight of the residue was calculated as follows: Weight of residue = (weight of dish + residue) − (weight of empty dish).

The total water extractive value was calculated as follows: percentage weight per weight

(1) $$\begin{array}{c} \;\left(\%\frac{w}{w}\right)=\frac{\text{weight of residue}}{\text{initial weight in }10\text{ mL}}\times 100. \end{array}$$

### 2.7. Microbial Load Test Using Total Bacterial, Fungal, and Pathogenic Bacterial Counts

The microbial safety and quality of the *L. multiflora* tea bags were assessed using the British Pharmacopoeia 2019 specifications. The tests were used to quantify the number of bacteria and fungi isolated that are able to grow aerobically in 1 g of sample. All microbial analyses were carried out in triplicate. Viability was assessed by the pour plate method using plate count agar for bacterial counts and malt extract agar for fungi identification.

The media were prepared according to the manufacturer's instructions and incubated at 37°C for 24 h for bacterial screening and at 25°C for 5 days for fungal screening. At the end of the incubation period, the number of colony-forming units per gram (CFU/g) was calculated by multiplying the average number of colonies by the dilution factor. All microbial analyses were carried out in triplicate. For investigating the presence of pathogenic bacteria, selective media used for the identification of *Escherichia coli, Staphylococcus spp.,* and *Salmonella spp.* were MacConkey agar, mannitol salt agar, and xylose lysine deoxycholate agar, respectively.

### 2.8. Mass Uniformity of the Formulated Tea Bags

The average mass of 20 randomly selected units of the tea bags was determined by weighing a single filled tea bag, opening it whilst making sure no fragments were lost with complete emptying. The weight of the empty bag was noted and the weight of the content was calculated by deduction. The procedure was repeated on the 19 remaining tea bags. The uniformity of mass was then determined. This procedure was done in triplicate for all four batches of products obtained from the three different processing methods [22].

### 2.9. Extraction Method Optimization

The method for optimization of the extraction was modified from Chandini et al. [26] and Yuto et al. [27]. One tea bag (2 g net weight) was soaked in 300 mL of hot water (freshly boiled), allowed to extract for about 10 minutes, and filtered. The volume of the filtrate was recorded and the total solid residue was determined. The total extract available in the filtrate was then calculated. The procedure was repeated using various volumes of the hot water (250, 200, and 150 mL) for the extraction procedure. The procedure was done in triplicate for each extraction volume. The same procedure above was also repeated using two tea bags (4 g net weight).

### 2.10. Statistical Analyses

The standard deviations and means of the replicate determinations were analyzed using Microsoft excel, 2016 version. The one-way analysis of variance (ANOVA) was used to analyze the pH, moisture content, and total water extractive data obtained from using the three different processing methods. GraphPad Prism version 7 was used to prepare all the plots. In the optimization of extraction study, linear regression analyses were employed.

## 3. Results and Discussion

### 3.1. Physicochemical Properties and Phytoconstituents Present in the Formulated Tea Bags

The results from the analyses of the four different batches of tea bags obtained using the three processing methods as presented in Table 1 showed that all samples produced by the three processing methods contained phytochemical constituents such as reducing sugars, phenolic compounds, flavonoids, alkaloids, saponins, polyuronides, anthracenosides, and phytosterols. The presence of these phytochemicals has been reported [1, 28] where the parameters were not affected by the drying methods used. A similar study was conducted by Manzano Santana et al. [21] where the drying methods used on Ilex guayusa leaves did not affect the phytochemical constituents present in the plant. It did, however, increase the concentration of secondary metabolites in the aqueous and ethanol extracts tested. Only alkaloids were found in the leaves of *L. multiflora* collected from six districts in Benin, according to Djengue et al. [29]. The current study validates the previous studies reported. The occurrence of these essential phytochemical constituents in *L. multiflora* may be contributed to the antifungal, antibacterial, antiedema, anti-inflammatory, antiviral, antimalarial, and anti-stress activities of the plant [28–33]. The results obtained indicate the oven temperature of 70°C did not affect the presence of the phytochemical constituents determined in the samples. The reduction in the amounts of volatile compounds in plant materials during oven-drying depends on the volatility and chemical structure of the constituents [34]. Radünz et al. [35] observed no significant difference in the essential oil content of fresh leaves of *L. sidoides* compared to the leaves that were oven-dried at temperatures of 40°C to 70°C. Meanwhile, the essential oil content of the sample dried in ambient air was 8% lower. When this evaluation was repeated, similar results were obtained with no significant qualitative changes in essential oils (thymol) comparable to the fresh plant [36]. *L. aliba* leaves were examined under six different drying treatments, using air at ambient temperature and air heated up to 80°C. The investigators concluded that drying of the plant for marketing purposes can be carried out using air that is heated to 40−80°C [37]. However, the changes in the chemical composition of essential oils and compounds present in herbal tea/products depend on individual medicinal plants, the drying temperature conditions, and the drying time [38, 39].

**Figure 1:**
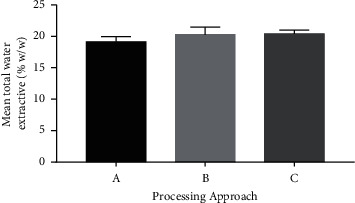
Graph of mean total water extractive from the various processing approaches of the formulated tea bags. Approach A: air-drying of the fresh leaves of *L. multiflora* Mold. before tea bagging; Approach B: oven-drying of the fresh leaves of *L. multiflora* Mold. before tea bagging; Approach C: microwaving of air-dried processed sample of *L. multiflora* Mold. before tea bagging. Values are mean ± SD.

The one-way ANOVA results for the total water extractives (means, standard deviation (SD) of the four different batches) after using the three different processing methods showed no significant difference (*P* > 0.05) in the means of the extractive values obtained, as has been indicated in Figure 1. This indicates that the different processing techniques did not affect the total water extractives. According to a study on the determination of total water extractives of poly-herbal formulations, the water-soluble extractives of the tested materials ranged from 8.23 to 34.52% [40]. From the results obtained as indicated in Figure 1, the total water extractive obtained was in the range of 19.53 to 20.93%, which indicates a good extraction for all three drying methods. A lower total extractive value may indicate an exhausted material, contamination, improper drying, storage, or preparation, whereas a higher total extractive value may also indicate the presence of more water-soluble contents in the plant material [40].

**Figure 2:**
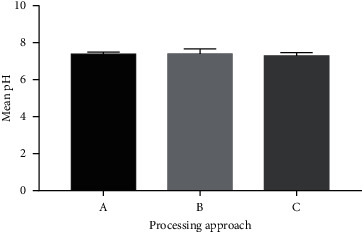
Graph of mean pH values obtained. Approach A: air-drying of the fresh leaves of *L. multiflora* Mold. before tea bagging; Approach B: oven-drying of the fresh leaves of *L. multiflora* Mold. before tea bagging; Approach C: microwaving of air-dried processed sample of *L. multiflora* Mold. before tea bagging. Values are mean ± SD.

The results (Figure 2) illustrated that the pH range of the products was 3.00 to 5.09. Statistical analysis of this result using the one-way ANOVA method of the samples prepared showed no significant difference (*P* > 0.05) in the pH values recorded for the different batches prepared via the different processing methods. This indicates that the processing methods did not affect the pH of the products. In a previous reported research project [41], the pH of some herbal medicines was determined to ascertain their acidity or alkalinity. Additionally, Onwordi et al. [42] have reported a pH range of 3.35–8.00 for some liquid herbal medicines sold in Nigeria, whereas an acidic pH range of 1.05–3.55 has also been reported for liquid herbal medicinal products [43]. Meanwhile, the obtained pH falls within the reported tolerable pH for natural plants, i.e., 4.0–7.5 [44]. Generally, the buffer systems of the body neutralize the accumulated acids and when this system is burdened, alkaline minerals from bones and vital organs are used by the body to neutralize acidic compounds. This results in the deterioration of the bones and the organs, leading to osteoporosis and undesirable effects on the immune, circulatory, digestive, respiratory, and other body systems [45]. The ingestion of herbal medicines with high acidic pH reduces the pH of the fluids in the stomach above the acceptable level required for metabolism, thus hindering enzyme action [45]. Enzymes are sensitive to pH changes and are known to function best at a specific pH range [45]. However, the pH values obtained for the *Lippia* tea bags from the three processing methods (A, B, and C) in this study ranged from 7.11 to 7.54, which is within the acceptable range of 4.0–7.5 reported by Edebi and Gideon [44]. This indicates that the teas produced from the various processing methods may be safe for consumption with regard to their pH.

**Figure 3:**
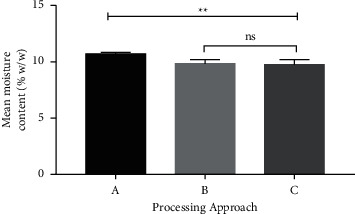
Graph of the mean moisture content. Approach A: air-drying of the fresh leaves of *L. multiflora* Mold. before tea bagging; Approach B: oven-drying of the fresh leaves of *L. multiflora* Mold. before tea bagging; Approach C: microwaving of air-dried processed sample of *L. multiflora* Mold. before tea bagging. Values are mean ± SD. *P* > 0.05 not significant (ns), *P* < 0.01 significant (^*∗∗*^).

The moisture content of the air-dried sample (A) was the highest, with an average value of 10.71%, followed by that of the oven-dried sample (B, 9.83%). The microwave-dried sample (C) had a moisture content of 9.75%. Nonetheless, statistical analysis using one-way ANOVA demonstrated no significant (P ˂ 0.01) difference between the recorded values for A compared to B and C as shown in Figure 3. This indicates that the processing methods affected the moisture contents of the products. A higher moisture content as seen in the air-dried sample may lead to increased susceptibility to microbial growth. Moisture content can be understood as the amount of water contained in a material or substance. The moisture content of a herbal plant helps to estimate the total solid matter and it is one of the major factors responsible for the deterioration of solid, powdered herbal drugs and formulations. A relatively lower moisture content is always desirable for higher drug stability and the prevention of fungal and bacterial growth. Eko et al. [46] in a moisture content determination study reported a low moisture content of 8.6% for the flowers, roots, leaves, fruits, and bark of medicinal plants that had been oven-dried, while the moisture content of air-dried samples was 9.81%. Ahmad et al. [47] have also reported moisture contents of 11.0% and 12.14% for field-grown leaves and roots, respectively, of *Rauvolfia serpentia* at 105°C. The moisture content of herbal plants depends on the nature and type of herb, postharvest practices, the age of the plants, and the chemical composition of the plant. The moisture contents of *L. multiflora* leaf, flower, and market powders, as evaluated by Djengue and his team [8], were found to be 7.42%, 9.89%, and 11.67%, respectively. The results of the present study showed that the moisture content of the *L. multiflora* leaves was in the range of 9.75–10.71%, which is consistent with the reported results [8].

### 3.2. Uniformity of Mass of the Formulated Tea Bags

For the uniformity of mass, the acceptance criteria for weights between 1.5 g and 2 g is that not more than two sachets should deviate by more than 10% from the average mass and none should deviate by more than 20% [22]. As can be observed from Table 2 the batches passed the uniformity of mass test. It can be inferred that the tea-bagging machine helped ensure the uniform filling of tea bags. In addition, it helps to provide a product that is convenient for patient usage compared to powders that have not been bagged.

### 3.3. Microbial Load of *L. multiflora* Mold. Tea Bags

Results of the microbial load analyses of the air-dried *L. multiflora* Mold. tea bags have been presented in Table 3. It can be observed that all the four batches of the products failed the microbial load test. The quality and safety of herbal teas for commercial use can be promoted if microbial contamination levels are addressed through appropriate quality control processes during the preparation of the plant material for tea bagging. Though some consumers believe that hot water steeping of herbal teas could reduce microbial contamination, once there is a high level of contamination beyond acceptable limits, steeping of herbal tea in hot or boiling water alone may not be able to clear the contamination to acceptable levels [48]. Drying plays a pivotal role in good manufacturing practices during the production of herbal products, as it helps reduce the moisture content of materials, thus preventing microbial contamination and prolonging the shelf life of the materials [49]. The presence of aerobic bacteria and fungi beyond acceptable limits may be due to frequent multiplication in the air under ambient temperature conditions and microbial contaminants settling on leaves during the drying process [50]. *E. coli* was present in the LTB1 and LTB3 samples. The presence of *E. coli* and *Staphylococcus spp.* has been reported in some homemade and commercial herbal medicine [51] as well as in some dried herbs and teas [52]. *E. coli* may cause urinary tract infections, pneumonia, and diarrhea [53]. Contamination from *Staphylococcus spp.* could also cause cellulitis, stomachache, scalded-skin syndrome, and impetigo [54]. The relatively higher moisture content of the air-dried powders (Figure 3) possibly resulted in the failing of the microbial load test. The high moisture content made the samples more susceptible to microbial contamination in addition to contamination from the environment during the air-drying process. A study by Djengue et al. [8] showed that high moisture and ash contents in the powders of *L. multiflora* favor the growth and development of yeast and molds. The load detection for *Salmonella spp.* in this study was within the acceptable criteria. Similar results have also been reported by Djengue and his team [8], who found the coliform detection threshold for *Salmonella spp.* in dried *L. multiflora* leaves to be below the acceptable limits.

From Table 4, it can be observed that the total aerobic bacteria, yeast, and mold counts in all the four batches of oven-dried *L. multiflora* Mold. leaves before tea bagging were within the acceptable specification limits, which could be due to the drying conditions employed. Kulshrestha et al. [55] have reported that drying herbs at a relatively high temperature decreases the total microbial counts. Research has also revealed that *L. multiflora* leaves contain some essential oils that have antimicrobial properties [13]. It has been revealed in some studies that essential oils in the leaves of plants have a strong inhibitory effect on the growth of *Staphylococcus aureus* and *Enterococcus hirae* and a moderate inhibitory effect on *Candida albicans* and *Saccharomyces cerevisiae* [30, 56, 57]. Specially, essential oils such as carvacrol and thymol have been shown to have antimicrobial properties in previous studies [32, 58, 59]. This may account for the low microbial contamination observed in *L. multiflora* tea bag samples investigated using oven-dried and microwave-dried methods. Meanwhile, high microbial contamination was observed in the air-dried samples.

It can be observed from Table 5 that tea bags produced from the microwaved air-dried samples passed the microbial load tests. In comparison to the air-dried sample, which failed the tests, it can be deduced that microwaving of the samples may have caused deactivation of the microbial agents present in the air-dried sample. Microwaves use nonionizing electromagnetic waves of 1 m to 1 mm and frequencies between 0.3 and 300 GHz [60]. The application of microwave treatment on spices and herb samples has been studied by Dababneh [61], who found that exposure of dry and wet spices and herbs to microwaves reduced microbial populations in the samples. The study concluded that decontamination of materials at home and in industries can be done by microwaving. The use of microwave radiation to reduce microbial contamination is applied in the pharmaceutical and food industries for drying, decontamination, defrosting, and inactivation of enzymes [62–66]. In addition, microwaving reduces the number of pathogenic and nonpathogenic bacteria in samples [67]. Research showed that microwave treatment reduced total bacteria coliforms and a yeast population (*Brettanomyces bruxellensis*) in oak wine barrels, as well as minimizing preservative use [68]. A study by Slobodan et al. [69] also revealed that microwave radiation can have substantial effects on the growth of microbial cultures. Additionally, the tendency to kill microorganisms depends on factors such as the frequency of the microwave and the tendency of microorganisms to absorb the microwave energy. However, high-energy and high-frequency microwaves prevent the growth of microorganisms. Thermal and nonthermal effects occur due to the radiation released from microwaving, which destroys the structure of the microorganisms [70]. In previous studies, the occurrence of thermal effects led to the absorption of radiation, which caused agitation of the molecules inside the bacterial cells to produce heat, thereby forming a mass in the cytoplasm due to protein denaturation [15, 70]. Nonthermal effects of microwaves, on the other hand, cause changes in the cell anatomy and proteins of microorganisms [70, 71]. Moreover, microwaving was reported to reduce *Salmonella* in peanut butter [72], and it was also used in drying plant-made products [73]. Microwaving also facilitates the extraction of biochemical compounds from plants via the drying process [74, 75]. Specifically, the process has been used in the isolation of essential oils and antioxidants within a short time from ethno-medicinal plants [76].

### 3.4. Optimization of Extraction of *Lippia tea* Bags

**Figure 4:**
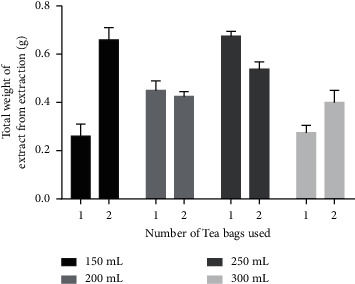
Weight of extracts obtained from the tea bags in the extraction procedure. “1” indicates one tea bag (2 g) and “2” indicates two tea bags (4 g).

It can be seen from Figure 4 and Table 6 that extraction was optimized with one tea bag in 250 mL of hot water for 10 minutes.

## 4. Conclusion

The approaches of oven-drying and microwaving of air-dried samples that were adopted to eliminate microbial contamination from tea bags prepared from *L. multiflora* leaves were successful. The moisture contents of these samples were relatively lower and the physiochemical properties were found to be within acceptable limits. The phytochemical constituents observed in the *L. multiflora* leaves were not affected by the applied methods. The oven and microwave drying methods caused a decrease in the level of microbial contamination in *L. multiflora* leaves to acceptable limits relative to the air-dried samples, making them safer for consumption relative to the air-dried sample. The optimized extraction procedure can be used to achieve an effective extraction using a minimal number of tea bags.

## Figures and Tables

**Table 1:** Phytochemical properties of the formulated tea bags.

Phytochemical test	Inference	Processing approach											
		A				B				C			
		LTBI	LTB2	LTB3	LTB4	LTBI	LTB2	LTB3	LTB4	LTBI	LTB2	LTB3	LTB4
Reducing sugar	Brick-red colouration	+	+	+	+	+	+	+	+	+	+	+	+
Saponins	Formation of froth which persists for 10 min	+	+	+	+	+	+	+	+	+	+	+	+
Phenolic compounds	Dark green colouration	+	+	+	+	+	+	+	+	+	+	+	+
Polyuronides	Violet precipitation	+	+	+	+	+	+	+	+	+	+	+	+
Flavonoids	Orange colouration	+	+	+	+	+	+	+	+	+	+	+	+
Anthracenosides	Red colouration	+	+	+	+	+	+	+	+	+	+	+	+
Alkaloids	Cream precipitation	+	+	+	+	+	+	+	+	+	+	+	+
Phytosterols	Dark green colouration	+	+	+	+	+	+	+	+	+	+	+	+

Approach A: air-drying of the fresh leaves of *L. multiflora* Mold. before tea bagging; Approach B: oven-drying of the fresh leaves of *L. multiflora* Mold. before tea bagging; Approach C: Microwaving of air-dried processed sample of *L. multiflora* Mold. before tea bagging. + means present.

**Table 2:** Uniformity of mass of the formulated tea bags.

Processing approach	Batch of product	Number of tea bags deviating by more than 10% from mean (*N* = 20)	Number of tea bags deviating by more than 20% from mean (*N* = 20)
A	LTB1	2	0
	LTB2	1	0
	LTB3	2	0
	LTB4	2	0

B	LTB1	1	0
	LTB2	2	0
	LTB3	1	0
	LTB4	1	0

C	LTB1	1	0
	LTB2	0	0
	LTB3	1	0
	LTB4	2	0

Approach A: air-drying of the fresh leaves of *L. multiflora* Mold. before tea bagging; Approach B: oven-drying of the fresh leaves of *L. multiflora* Mold. before tea bagging; Approach C: microwaving of air-dried processed sample of *L. multiflora* Mold. before tea bagging.

**Table 3:** Microbial load analyses of tea bags prepared from the air-dried leaves of *L. multiflora* Mold.

Test conducted	LTB1 (cfu/g)	LTB2 (cfu/g)	LTB3 (cfu/g)	LTB4 (cfu/g)	Acceptance criterion (BP, 2019)
^1^TAMC/37 °C/24 hours/PCA	8.6 × 10^8^	TNTC	TNTC	7.3 × 10^9^	≤5.0 × 10^7^
^2^TYMC/25 °C/5 days/MEA	4.9 × 10^7^	TNTC	TNTC	5.1 × 10^6^	≤5.0 × 10^5^
*Escherichia coli (E. coli)*	+	−	+	−	Absence
*Salmonella spp.*	−	−	−	−	Absence
*Staphylococcus spp*.	−	+	−	+	Absence

BP, British Pharmacopoeia; ^1^TAMC: total aerobic microbial counts, ^2^TYMC: total yeast and mold counts; TNTC: too numerous to count; −: absent; +: present; PCA: plate count agar; MEA: malt extract agar.

**Table 4:** Microbial load analyses of tea bags prepared from the oven-dried leaves of *L. multiflora* Mold.

Test conducted	LTB1 (cfu/g)	LTB2 (cfu/g)	LTB3 (cfu/g)	LTB4 (cfu/g)	Acceptance criterion (BP, 2019)
^1^TAMC/37°C/24 h/PCA	1.2 × 10^3^	1.1 × 10^3^	1.8 × 10^2^	2.5 × 10^4^	≤5.0 × 10^7^
^2^TYMC/25°C/5 days/MEA	6.0 × 10^2^	5.5 × 10^2^	3.9 × 10^3^	7.4 × 10^4^	≤5.0 × 10^5^
*E. coli*	−	−	−	−	Absence
*Salmonella spp.*	−	−	−	−	Absence
*Staphylococcus spp.*	−	−	−	−	Absence

BP, British Pharmacopoeia; ^1^TAMC: total aerobic microbial counts; ^2^TYMC: total yeast and mold counts; −: absent; +: present; PCA: plate count agar; MEA: malt extract agar.

**Table 5:** Microbial load analyses of tea bags prepared from the microwaved air-dried leaves of *L. multiflora* Mold.

Test conducted	LTB1 (cfu/g)	LTB2 (cfu/g)	LTB3 (cfu/g)	LTB4 (cfu/g)	Acceptance criterion (BP, 2019)
^1^TAMC/37°C/24 h/PCA	8.7 × 10^4^	1.3 × 10^3^	2.6 × 10^4^	6.9 × 10^5^	≤5.0 × 10^7^
^2^TYMC/25°C/5 days/MEA	6.5 × 10^3^	9.1 × 10^4^	4.3 × 10^3^	3.8 × 10^3^	≤5.0 × 10^5^
*E. coli*	−	−	−	−	Absence
*Salmonella spp.*	−	−	−	−	Absence
*Staphylococcus spp.*	−	−	−	−	Absence

BP, British Pharmacopoeia; ^1^TAMC: total aerobic microbial counts; ^2^TYMC: total yeast and molds counts; −: absent; +: present; PCA: plate count agar; MEA: malt extract agar.

**Table 6:** Results of optimization of extraction from *Lippia* tea bags.

Weight of tea bags used in extraction (g)	Volume of water used in extraction (mL)	Volume of filtrate obtained (mL) *N* = 3	Total solid residue (% w/v) *N* = 3	Total extract in filtrate (g)
2	300	265 ± 5	0.15 ± 0.02	0.400 ± 0.05
2	250	215 ± 2	0 .25 ± 0.01	0.538 ± 0.02
2	200	170 ± 5	0.25 ± 0.02	0.425 ± 0.03
2	150	130 ± 2	0.20 ± 0.04	0.26 ± 0.05
4	300	275 ± 4	0.10 ± 0.01	0.275 ± 0.03
4	250	225 ± 3	0.30 ± 0.01	0.675 ± 0.02
4	200	180 ± 5	0.25 ± 0.02	0.450 ± 0.04
4	150	120 ± 2	0.55 ± 0.04	0.660 ± 0.05

## Data Availability

The data used to support the findings of this study are available from the corresponding author upon request.
